# Examining the Relationship Between Dietary Glycemic Load (GL), Glycemic Index (GI), and the Odds of Diminished Ovarian Reserve: A Case–Control Study

**DOI:** 10.1002/fsn3.70515

**Published:** 2025-07-24

**Authors:** Abed Ghavami, Mahdieh Khodarahmi, Sanaz Mehrabani, Amin Mokari‐Yamchi, Hatav Ghasemi‐Tehrani, Mahdi Vajdi, Gholamreza Askari

**Affiliations:** ^1^ Department of Clinical Nutrition, School of Nutrition and Food Sciences Isfahan University of Medical Sciences Isfahan Iran; ^2^ Nutrition and Food Security Research Center Isfahan University of Medical Sciences Isfahan Iran; ^3^ Maternal and Childhood Obesity Research Center Urmia University of Medical Sciences Urmia Iran; ^4^ Department of Obstetrics and Gynecology, School of Medicine Isfahan University of Medical Sciences Isfahan Iran; ^5^ Department of Community Nutrition, School of Nutrition and Food Science, Nutrition and Food Security Research Center Isfahan University of Medical Sciences Isfahan Iran

**Keywords:** dietary glycemic index, dietary glycemic load, diminished ovarian reserve

## Abstract

A decline in both the number and quality of oocytes within the ovaries indicates a reduced ovarian reserve. As lifestyle factors, particularly dietary habits, can influence ovarian reserve, this study aimed to assess the relationship between dietary glycemic load (GL) and glycemic index (GI) and odds of diminished ovarian reserve (DOR). The current case–control study includes 370 women, consisting of 120 women who had DOR and 250 control participants. The two groups were matched based on age and body mass index (BMI). The dietary information was collected using a semiquantitative food frequency questionnaire (FFQ) that consisted of 80 items. A multivariable logistic regression approach was used to assess the association of dietary GI and GL scores with the odds of DOR, adjusting for potential confounders. The initial result revealed that there was no significant association between the odds of DOR and the dietary GI and GL when assessed through both crude and adjusted analyses, which accounted for factors such as physical activity and energy intake. However, when adjustments were made for factors such as physical activity, energy intake, fat mass (FM), and BMI, it was observed that women who were in the highest quartile of the dietary GL and GI scores had a 13% (OR 1.13; 95% CI 1.07–2.68, *p* = 0.042) and 19% (OR 1.19; 95% CI 0.59–1.87, *p* = 0.038) higher odds of DOR, respectively. Higher dietary GL and GI may be associated with increased odds of DOR. However, it is essential to conduct further studies with prospective design to establish this relationship and explore potential underlying mechanisms.

## Graphical abstract

The higher dietary glycemic index (GI) and glycemic load (GL) were significantly associated with indicators of diminished ovarian reserve (DOR), including reduced antral follicle count (AFC) and increased odds of DOR. These associations persisted even after controlling for key confounding variables such as physical activity, energy intake, fat mass (FM), and BMI.

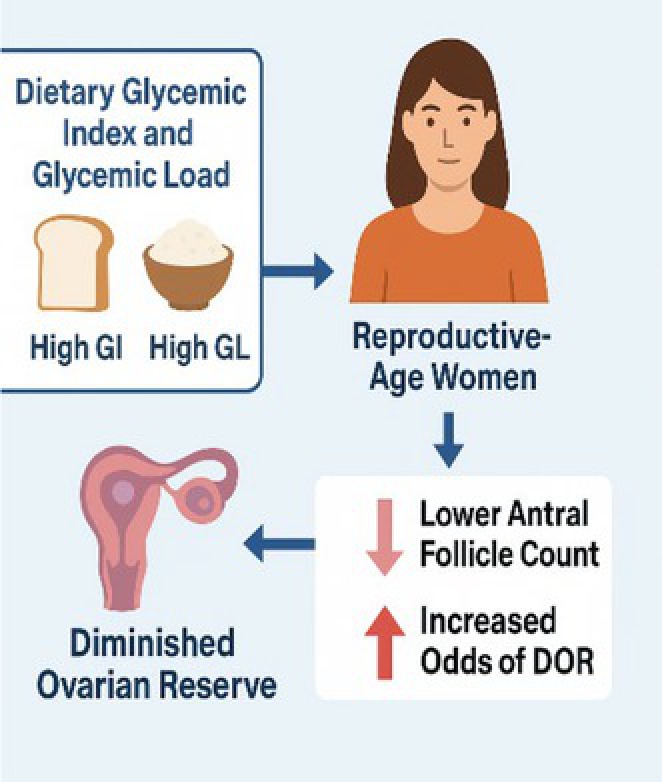

## 1 Introduction

Currently, nearly 17.5% of couples suffer from infertility worldwide and the cases have increased over the previous years (Cox et al. 2022). The decrease in ovarian reserve, also known as diminished ovarian reserve (DOR), is one of the factors contributing to female infertility. Some studies have revealed that lifestyle factors such as diet can influence ovarian function (Jiang et al. 2023; Génard‐Walton 2023; Eskew et al. 2022; Ziaei et al. 2023). Studies have demonstrated that following certain dietary patterns can improve reproductive outcomes (Eskew et al. 2022; Yang et al. 2023). However, previous studies have not adequately examined the impact of diet as a modifiable factor on ovarian reserve. This gap highlights the need for further research to clarify how dietary choices may influence ovarian function.

A key area of interest is the role of dietary carbohydrate intake, both as a primary energy source and as a potential modulator of fertility. Limited research has investigated the relationship between carbohydrate intake, both in quantity and type, and markers of ovarian reserve (Mitsunami et al. 2025). One study revealed that carbohydrate sources from dairy foods can positively influence ovarian reserve by slowing the decline in anti‐Mullerian hormone levels (Moslehi et al. 2019). Moreover, animal studies have indicated that diets high in refined carbohydrate can negatively affect the ovarian follicular reserve, possibly by triggering inflammation, oxidative stress, and insulin resistance (Niño et al. 2020). The glycemic index (GI) and glycemic load (GL) are dietary indices that reflect the physiological effects of carbohydrate intake. The GI is a measure used to evaluate how quickly and significantly a carbohydrate‐containing food raises postprandial blood glucose levels. Foods with a higher GI cause a more pronounced enhancement in postprandial blood glucose levels due to the rapid absorption of glycemic carbohydrates. The GL, which includes the GI and the amount of carbohydrates, provides a more comprehensive assessment of the effect of a diet on blood glucose and insulin responses (Jayedi et al. 2022).

Previous studies have shown that women consuming a high GI and GL foods are at greater odds of experiencing infertility (Aghaei et al. 2023). Therefore, this suggests that dietary factors related to glucose and insulin dynamics may impact ovarian reserve. Diets rich in high GI or load can increase insulin resistance, systemic inflammation, and oxidative stress (Galarregui et al. 2018; Manta et al. 2023; Anderson, Milne, et al. 2018) which may have downstream effects on ovarian function (Huang et al. 2023). Therefore, dietary indices related to glucose and insulin regulation may play a role in ovarian reserve status.

Although the biological rationale indicates a positive connection between GI and GL with insulin resistance that negatively influences ovarian function, the dietary GI and GL's associations with DOR remain unknown. As there was a gap in this area, therefore, more studies are needed to assess the association between dietary glycemic indices and ovarian health. This study was conducted using a case–control design among women attending infertility clinics to evaluate the potential link between dietary GI/GL and the odds of DOR. The findings may contribute to our knowledge about the role of dietary intake on women's reproductive health.

## 2 Materials and Methods

### 2.1 Participants

A total of 370 Iranian women including 120 subjects with DOR and 250 age‐ and BMI‐matched controls were enrolled in this case–control study. All participants were between the ages of 18 and 45 years and had a BMI ranging from 20 to 35 kg/m^2^. The study included subjects in different age groups (under 25, 25–30, and over 30 years old) and BMI categories (under 24.9, 25–30, and over 30 kg/m^2^). Participants were recruited from infertility centers affiliated with Isfahan University of Medical Sciences in Iran through purposive sampling. DOR was diagnosed by a qualified gynecologist (H.G.‐T.) based on either low AMH levels (≤ 0.7 ng/mL), a low AFC (≤ 4 in both ovaries), or both criteria (Penzias et al. 2020). Controls with normal ovarian reserve were randomly selected from the same centers and matched to DOR cases based on age and BMI categories.

The sample size calculation was conducted using the standard case–control formula, based on a type I error of 0.05 (*α* = 0.05), a 95% confidence interval, and 90% power (*β* = 0.10). According to the previous evidence, around 30% of healthy women had high dietary GI or GL (Moridi et al. 2020; Mitsunami et al. 2023). For sample size estimation, an odds ratio (OR) of 2.0 was assumed for the association between elevated GI/GL and DOR. Consequently, the minimum required sample sizes for the case and control groups were estimated to be 120 and 250 participants, respectively.

**FIGURE 1:**
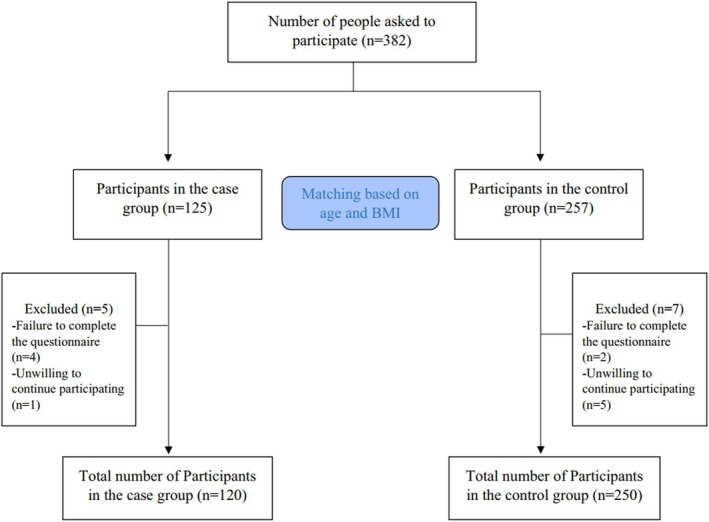
Flow chart of the study.

Participants were not eligible for inclusion if they had undergone ovarian surgery, chemotherapy, or radiotherapy. Additional exclusion criteria included conditions such as endometriosis, premature ovarian failure, and various endocrine or metabolic disorders. Additionally, individuals were also excluded if they had received hormone therapy, followed specific diets, or utilized oral contraceptives within the 3 months prior to the study commencement. Participants with substantial overreporting and underreporting for dietary intakes and those with chronic diseases such as cancers, cardiovascular diseases (CVDs), liver failure, and autoimmune diseases were excluded as well. All subjects provided signed informed consent prior to participation. The research protocol was approved by the Ethics Committee of Isfahan University of Medical Sciences (IR.ARI.MUI.REC.1401.297). The study process is visually represented in the flowchart in Figure 1.

### 2.2 Dietary Assessment

In the current study, participants' dietary intakes were collected using a validated and reproducible 80‐item semiquantitative food frequency questionnaire (FFQ) (Nikniaz et al. 2017). An expert dietitian conducted face‐to‐face interviews with all participants to complete the FFQs. Portion sizes for all food items were converted to grams using standard Iranian household measures (Ghaffarpour et al. 1999). To evaluate the energy, macronutrients, and micronutrients derived from participants' dietary intakes, the modified version of Nutritionist IV software for Iranian foods was utilized (Azar and Sarkisian 1980).

### 2.3 Carbohydrate Indices

For each carbohydrate‐containing food, the GI is defined as the area under the blood glucose response curve over 2 h following consumption of that food, compared to the response after ingesting an equivalent amount of carbohydrates as glucose (Jenkins et al. 1981). To determine the GI value for each food item, we utilized the international GI table and a list of the GI values for Iranian foods (Foster‐Powell et al. 2002; Taleban and Esmaeili 1999). The total dietary GI and GL were then calculated as follows:

$$\begin{array}{cc} \text{Dietary}\;\mathrm{GI}\; & =[\left(\text{carbohydrate content of each food item}\right)\\ & \times \left(\text{number of servings}/d\right)\times \left(\text{glycemic index}\right)]/\\ & \text{total daily carbohydrate intake} \end{array}$$

$$\begin{array}{cc} \text{Dietary}\;\mathrm{GL}\; & =\left(\text{carbohydrate content of each food item}\right)\\ & \times \left(\text{number of servings}/d\right)\times \left(\mathrm{GI}\right) \end{array}$$

### 2.4 Anthropometric and Laboratory Assessments

Participants' weight was measured with minimal clothing and without shoes, rounded to the nearest 100 g. Height measurements were taken to the nearest 0.5 cm. Body mass index (BMI) was calculated by dividing weight in kilograms by the square of height in meters. Waist circumference (WC) and hip circumference (HC) were measured using a tape measure to the nearest 0.1 cm. WC was assessed at the midpoint between the lowest rib and the iliac crest, while HC was measured at the widest part of the buttocks. The waist‐to‐hip ratio (WHR) was determined by dividing WC by HC. To evaluate body composition, including fat mass (FM) and fat‐free mass (FFM), bioelectrical impedance analysis (BIA) was conducted using a body composition analyzer (Inbody 770; Inbody Co, Seoul, Korea). The physical activity level of participants was assessed using the International Physical Activity Questionnaire (IPAQ). The validation of this questionnaire has previously been done for the Iranian population (Moghaddam et al. 2012). Serum AMH levels were measured using the enzyme‐linked immunosorbent assay (ELISA) method (Monobind, California, USA). Furthermore, a transvaginal ultrasound was conducted to evaluate the total antral follicle count (AFC) in both ovaries on the third day of an unstimulated menstrual cycle. Systolic and diastolic blood pressure (SBP and DBP) were measured twice on the right arm of seated participants using an automated digital sphygmomanometer (Microlife Blood Pressure Monitor A100‐30; Berneck, Switzerland) after a 15‐min period of rest in a relaxed position. The average of the two readings was recorded as the participant's blood pressure.

### 2.5 Statistical Analyses

The normality of the variables was assessed using the Kolmogorov–Smirnov test and histogram charts. Baseline characteristics of participants were presented as mean ± standard deviation (SD) for quantitative variables and as frequencies (percentages) for qualitative variables. Independent sample *t*‐test and chi‐square test were used to analyze the differences between cases and controls for continuous and categorical variables, respectively. Participants were classified into quartiles based on dietary GI and GL scores. A one‐way ANOVA was employed to evaluate differences in continuous variables, while a chi‐square test was used for categorical variables across the quartiles of the GI and GL scores. To explore the relationship between GI and GL scores and the odds of DOR, multivariable logistic regression was performed using two models with multiple covariates. Potential confounding variables were chosen based on existing literature (Eskew et al. 2022) and a Directed Acyclic Graph (Rothman et al. 2008). Model I was adjusted for physical activity and energy intake, while Model II included additional adjustments for FM and BMI. All analyses were conducted using the Statistical Package for the Social Sciences (SPSS) (version 21.0; SPSS Inc., Chicago, Illinois, USA).

## 3 Results

Data was collected from 250 participants in the control group and 120 participants in the DOR group. However, seven participants from the control group and five from the DOR group were excluded from the final analysis—six for not completing the questionnaire and six for unwillingness to continue participating in the study (Figure 1). General characteristics of the study participants are provided in Table 1. The mean (SD) age of participants was 33.37 (3.24) and 32.91 (3.15) years in cases and controls, respectively, demonstrating the frequency‐matching design. DOR patients had significantly higher FM (38.47 vs. 36.47), WC (102.23 vs. 91.7), and WHR (0.9 vs. 0.86). Additionally, serum levels of AMH (0.56 vs. 4.11) and AFC count (2.34 vs. 9.59) were significantly lower in the DOR group compared to the control group (*p* < 0.05).

**TABLE 1:** Baseline characteristic of participants.

Variable		Case (*N* = 120)	Control (*N* = 250)	*p* ^a^
Age (years)		33.37 ± 3.24	32.91 ± 3.15	0.196
BMI (kg/m^2^)		29.85 ± 2.49	27.75 ± 3.45	0.235
Weight (kg)		80.96 ± 4.78	79.26 ± 8.41	0.487
FM (kg)		38.47 ± 7.05	36.47 ± 8.91	0.020
FFM (kg)		57.99 ± 11.33	60.12 ± 11.97	0.098
WC (cm)		102.23 ± 35.95	91.70 ± 12.43	0.002
HC (cm)		109.10 ± 31.59	106.10 ± 11.57	0.316
WHR		0.90 ± 0.12	0.86 ± 0.08	0.003
SBP (mmHg)		122.18 ± 12.77	123.58 ± 14.03	0.341
DBP (mmHg)		79.41 ± 11.67	81.85 ± 10.48	0.056
Physical activity (MET/h/day)		19.05 ± 4.12	18.98 ± 4.51	0.896
Socioeconomic status (SES) (%)	Low	10 (8.3)	19 (7.6)	0.252
	Middle	50 (41.7)	127 (50.8)	
	High	60 (50)	104 (41.6)	
Education (%)	Illiterate	14 (11.7)	34 (13.6)	< 0.001
	≤ High school/diploma	31 (25.8)	121 (48.4)	
	≥ College degree	75 (62.5)	95 (38)	
Occupation (%)	Housewife	82 (68.3)	184 (73.6)	< 0.001
	Employed	26 (21.7)	10 (4)	
	Student	12 (10)	56 (22.4)	
Pervious Pregnancy	Yes	99 (82.5)	203 (81.2)	0.441
	No	21 (17.5)	47 (18.8)	
AFC count		2.34 ± 1.19	9.59 ± 2.24	< 0.001
AMH (ng/mL)		0.56 ± 0.71	4.11 ± 1.18	< 0.001

*Note:* Quantitative variables are expressed as mean ± SD and qualitative variables expressed as *n* (%).

Abbreviations: AFC, antral follicle count; AMH, anti‐Müllerian hormone; BMI, body mass index; DBP, diastolic blood pressure; DOR duration, diminished or decreased ovarian reserve FFM, fat free mass; FM, fat mass; HC, hip circumference; SBP, systolic blood pressure; WC, waist circumference; WHR, waist to hip ratio.

^a^ *p* values resulted from independent *t*‐tests for quantitative and Chi‐square for qualitative variables between the two groups.

Tables 2 and 3 presented the characteristics of the study participants divided into quartiles based on their GL and GI scores. Participants in the highest quartile of GL and GI had significantly lower AFC count (*p* = 0.001) and higher weight (*p* = 0.032) compared to those in the lowest quartile. Furthermore, women with DOR showed significantly higher SBP associated with elevated GI scores (*p* = 0.002). However, there were no significant differences in other variables, including age, BMI, FM, WC, WHR, physical activity, and socioeconomic status across the quartiles of dietary GL and GI. The energy, nutrient, and food group intakes of study participants across quartiles of the GL and GI are presented in Tables 4 and 5.

**TABLE 2:** Characteristics of study participants according to quartiles of dietary glycemic load (DGL).

Variable		Case (120)					Control (250)				
		Q1	Q2	Q3	Q4	*p* ^a^	Q1	Q2	Q3	Q4	*p* ^a^
Age (years)		33.95 ± 3.30	32.40 ± 3.39	33.36 ± 2.53	33.65 ± 3.57	0.314	32.96 ± 3.11	32.50 ± 3.27	33.44 ± 3.08	32.65 ± 3.13	0.379
BMI (kg/m^2^)		29.72 ± 2.41	30.46 ± 2.54	29.45 ± 2.25	29.68 ± 2.78	0.545	28.05 ± 3.41	28.00 ± 2.80	26.96 ± 3.75	28.02 ± 3.67	0.205
Weight (kg)		82.12 ± 4.27	81.65 ± 3.77	81.50 ± 3.09	84.33 ± 4.73	0.032	78.45 ± 4.21	77.70 ± 5.19	78.20 ± 5.04	78.27 ± 5.18	0.857
FM (kg)		38.07 ± 5.60	37.76 ± 5.59	39.62 ± 6.95	38.39 ± 9.32	0.764	36.06 ± 8.66	37.38 ± 9.31	36.01 ± 9.83	36.84 ± 7.89	0.306
FFM (kg)		58.87 ± 11.73	56.57 ± 11.66	57.85 ± 11.31	58.46 ± 11.10	0.883	60.99 ± 12.48	57.53 ± 10.43	61.35 ± 12.67	60.48 ± 12.02	0.276
WC (cm)		100.09 ± 35.05	104.33 ± 41.86	107.86 ± 35.31	97.25 ± 32.73	0.675	91.81 ± 12.12	90.25 ± 11.43	93.75 ± 13.08	90.87 ± 12.94	0.413
HC (cm)		105.09 ± 25.25	113.66 ± 35.43	110.06 ± 30.84	108.21 ± 35.16	0.778	106.31 ± 12.82	105.36 ± 11.67	107.90 ± 11.44	104.76 ± 10.33	0.443
WHR		0.90 ± 0.15	0.90 ± 0.09	0.90 ± 0.11	0.89 ± 0.12	0.993	0.86 ± 0.07	0.85 ± 0.07	0.87 ± 0.10	0.86 ± 0.07	0.853
SBP (mmHg)		115.25 ± 11.62	123.88 ± 13.26	123.53 ± 12.03	122.71 ± 11.62	0.122	122.70 ± 14.56	121.66 ± 13.10	125.84 ± 14.43	123.90 ± 13.95	0.381
DBP (mmHg)		77.29 ± 10.92	83.03 ± 11.26	78.40 ± 11.94	79.37 ± 12.29	0.280	81.63 ± 11.16	80.33 ± 11.19	82.46 ± 10.46	82.87 ± 10.67	0.580
AFC count		2.70 ± 0.90	2.80 ± 0.99	1.71 ± 1.25	2.14 ± 1.32	0.001 0.00	10.45 ± 2.13	10.01 ± 2.45	9.38 ± 2.00	8.44 ± 1.83	< 0.001
AMH (ng/mL)		0.72 ± 1.36	0.52 ± 0.20	0.47 ± 0.23	0.52 ± 0.20	0.530	4.42 ± 1.06	4.30 ± 1.26	3.93 ± 1.11	3.80 ± 1.21	0.011
Physical activity (MET/h/day)		19.00 ± 4.57	19.48 ± 3.80	19.50 ± 4.32	19.25 ± 3.84	0.827	19.75 ± 4.31	19.28 ± 4.71	18.50 ± 4.69	18.46 ± 4.29	0.310
Socioeconomic status (SES) (%)	Low	2 (6.5)	4 (14.8)	2 (6.7)	2 (6.3)	0.699	8 (13.1)	3 (5)	3 (4.6)	5 (7.8)	0.553
	Middle	13 (41.9)	11 (40.7)	10 (33.3)	16 (50)		31 (50.8)	33 (55)	32 (49.2)	31 (48.4)	
	High	16 (51.6)	12 (44.44)	18 (60)	14 (43.8)		22 (36.1)	24 (40)	30 (46.2)	28 (43.8)	
Education (%)	Illiterate	3 (9.7)	3 (11.1)	5 (16.7)	3 (9.4)	0.347	7 (11.5)	10 (16.7)	12 (18.5)	5 (7.8)	0.527
	≤ High school/diploma	4 (12.9)	6 (22.2)	10 (33.3)	11 (34.4)		31 (50.8)	27 (45)	27 (41.5)	36 (56.3)	
	≥ College degree	24 (77.4)	18 (66.7)	15 (50)	18 (56.3)		23 (37.7)	23 (38.3)	26 (40)	23 (35.9)	
Occupation	Housewife	19 (61.3)	19 (70.4)	23 (76.7)	21 (65.6)	0.910	44 (72.1)	46 (76.7)	46 (70.8)	48 (75)	0.901
	Employed	8 (25.8)	5 (18.5)	5 (16.7)	8 (25)		2 (3.3)	1 (1.7)	4 (6.2)	3 (4.7)	
	Student	4 (12.9)	3 (11.1)	2 (6.7)	3 (9.4)		15 (24.6)	13 (21.7)	15 (23.1)	13 (20.3)	
Pervious Pregnancy	No	27 (87.1)	22 (81.5)	21 (70)	29 (90.6)	0.159	50 (82)	53 (88.3)	52 (80)	48 (75)	0.296
	Yes	4 (12.9)	5 (18.5)	9 (30)	3 (9.4)		11 (18)	7 (11.7)	13 (20)	16 (25)	

*Note:* Quantitative variables are expressed as mean ± SD and qualitative variables expressed as *n* (%). The SES scored was evaluated based on education level of both subjects and the family head, job of both subjects and the family head family size, home status and home type by using self‐reported questionnaire.

Abbreviations: AFC, antral follicle count; BMI, body mass index; DBP, diastolic blood pressure; DOR duration, diminished or decreased ovarian reserve FFM, fat free mass; FM, fat mass; HC, hip circumference; SBP, systolic blood pressure; WC, waist circumference; WHR, waist to hip ratio.

^a^ *p* values resulted from ANOVA test for quantitative and Chi‐square test for qualitative variables across quartiles.

**TABLE 3:** Characteristic of study participants according to quartiles of dietary glycemic index (DGI).

Variable		Case (120)					Control (250)				
		Q1	Q2	Q3	Q4	*p* ^a^	Q1	Q2	Q3	Q4	*p* ^a^
Age (years)		33.93 ± 3.05	32.40 ± 3.39	33.36 ± 2.53	33.65 ± 3.57	0.314	32.96 ± 3.11	32.55 ± 3.27	33.44 ± 3.08	32.65 ± 3.13	0.379
BMI (kg/m^2^)		29.72 ± 2.41	30.46 ± 2.54	29.59 ± 2.25	29.68 ± 2.78	0.545	28.05 ± 3.41	28.00 ± 2.80	26.96 ± 3.75	28.02 ± 3.67	0.205
Weight (kg)		81.50 ± 3.09	81.65 ± 3.77	82.12 ± 4.27	84.33 ± 4.73	0.032	78.45 ± 4.21	77.70 ± 5.19	78.22 ± 5.04	78.28 ± 5.18	0.857
FM (kg)		38.07 ± 5.60	37.76 ± 5.59	39.62 ± 6.95	38.39 ± 9.32	0.764	36.06 ± 8.66	37.38 ± 9.31	36.01 ± 9.83	36.48 ± 7.89	0.821
FFM (kg)		58.87 ± 11.73	56.57 ± 11.66	57.85 ± 11.31	58.46 ± 11.10	0.883	60.99 ± 12.48	57.53 ± 10.43	61.35 ± 12.67	60.48 ± 12.05	0.276
WC (cm)		100.09 ± 35.05	104.33 ± 41.86	107.86 ± 35.31	97.25 ± 32.73	0.675	91.81 ± 12.12	90.25 ± 11.41	93.75 ± 13.08	104.76 ± 10.33	0.413
HC (cm)		105.09 ± 25.25	113.66 ± 35.43	110.06 ± 30.84	108.21 ± 35.16	0.778	106.31 ± 12.82	105.36 ± 11.67	107.90 ± 11.44	104.76 ± 10.33	0.443
WHR		0.90 ± 0.15	0.90 ± 0.09	0.90 ± 0.11	0.89 ± 0.12	0.993	0.86 ± 0.07	0.85 ± 0.07	0.87 ± 0.10	0.86 ± 0.07	0.853
SBP (mmHg)		115.35 ± 11.62	122.71 ± 11.62	123.53 ± 12.03	127.88 ± 13.26	0.002	122.70 ± 14.56	121.66 ± 13.10	125.84 ± 14.43	123.90 ± 13.95	0.381
DBP (mmHg)		77.29 ± 10.92	83.03 ± 11.26	78.40 ± 11.94	79.37 ± 12.29	0.280	81.63 ± 11.16	80.33 ± 11.19	82.46 ± 10.46	82.87 ± 10.67	0.580
AFC count		2.80 ± 0.99	2.70 ± 0.90	2.14 ± 1.32	1.71 ± 1.25	0.001	10.45 ± 2.13	10.01 ± 2.45	9.38 ± 2.00	8.44 ± 1.83	0.001>
AMH (ng/mL)		0.72 ± 1.36	0.52 ± 0.20	0.47 ± 0.23	0.52 ± 0.20	0.530	4.30 ± 1.26	4.20 ± 1.06	3.93 ± 1.11	3.80 ± 1.21	0.011
Physical activity (MET/h/day)		19.00 ± 4.57	19	18.50 ± 4.32	19.25 ± 3.84	0.764	19.75 ± 4.31	19.28 ± 4.71	18.50 ± 4.69	18.48 ± 4.29	0.310
Socioeconomic status (SES) (%)	Low	4 (12.9)	1 (3.3)	3 (10.3)	2 (6.7)	0.658	6 (9.4)	4 (6.3)	5 (8.3)	4 (6.3)	0.416
	Middle	13 (41.9)	13 (43.3)	9 (31)	15 (50)		29 (45.3)	40 (63.5)	27 (45)	31 (49.2)	
	High	14 (45.2)	16 (53.3)	16 (58.6)	13 (43.3)		29 (45.3)	19 (30.2)	28 (46.7)	28 (44.4)	
Education (%)	Illiterate	2 (6.5)	6 (20)	4 (13.8)	2 (6.7)	0.426	12 (18.8)	13 (20.6)	4 (6.7)	5 (7.9)	0.172
	≤ High school/diploma	8 (25.8)	7 (23.3)	10 (34.5)	6 (20)		29 (45.3)	30 (47.6)	29 (48.3)	33 (52.4)	
	≥ College degree	21 (67.7)	17 (56.7)	15 (41.7)	22 (73.3)		23 (35.9)	20 (31.7)	27 (45)	25 (39.7)	
Occupation	Housewife	20 (64.5)	20 (66.7)	21 (72.4)	21 (70)	0.992	48 (75)	45 (71.4)	44 (73.3)	47 (74.6)	0.953
	Employed	7 (22.6)	7 (23.3)	6 (20)	6 (20)		3 (4.7)	3 (4.8)	1 (1.7)	3 (4.8)	
	Student	4 (12.9)	3 (10)	22 (73.3)	3 (10)		13 (20.3)	15 (23.8)	15 (25)	13 (20.6)	
Pervious Pregnancy	No	28 (90.3)	26 (86.7)	22 (75.9)	23 (76.7)	0.352	50 (78.1)	53 (84.1)	48 (80)	52 (82.5)	0.830
	Yes	3 (9.7)	4 (13.3)	7 (24.1)	7 (23.3)		14 (21.9)	10 (15.9)	12 (20)	11 (17.5)	

*Note:* Quantitative variables are expressed as mean ± SD and qualitative variables expressed as *n* (%). The SES scored was evaluated based on education level of both subjects and the family head, job of both subjects and the family head family size, home status and home type by using self‐reported questionnaire.

Abbreviations: AFC, antral follicle count; BMI, body mass index; DBP, diastolic blood pressure; DOR, duration diminished or decreased ovarian reserve; FFM, fat free mass; FM, fat mass; HC, hip circumference; SBP, systolic blood pressure; WC, waist circumference; WHR, waist to hip ratio.

^a^ *p* values resulted from ANOVA test for quantitative and Chi‐square test for qualitative variables across quartiles.

**TABLE 4:** Energy, nutrient, and food group intakes of study participants across quartiles of the dietary glycemic load (DGL).

Variable	Case (120)					Control (250)				
	Q1	Q2	Q3	Q4	*p* ^a^	Q1	Q2	Q3	Q4	*p* ^a^
Energy (Kcal/day)	2452.94 ± 740.01	2394.31 ± 627.33	2617.39 ± 824.12	2870.34 ± 887.06	< 0.001	2375.32 ± 542.68	2483.23 ± 602.37	2583.63 ± 702.31	2883.92 ± 702.21	< 0.001
Carbohydrate (g/day)	328.36 ± 128.11	340.87 ± 128.57	385.36 ± 140.79	398.21 ± 144.62	< 0.001	342.12 ± 103.52	378.65 ± 115.42	394.89 ± 113.17	425.27 ± 118.19	< 0.001
Fat (g/day)	78.33 ± 32.27	79.66 ± 22.81	80.42 ± 36.68	86.72 ± 30.85	< 0.001	85.08 ± 31.10	88.79 ± 32.68	90.21 ± 32.10	94.24 ± 33.71	< 0.001
Protein (g/day)	81.9 ± 31.94	79.1 ± 29.89	90.8 ± 29.51	96.8 ± 28.24	< 0.001	94.95 ± 29.51	98.61 ± 31.27	99.24 ± 34.42	104.84 ± 33.82	< 0.001
Total fiber (g/day)	18.63 ± 11.71	17.92 ± 8.38	18.31 ± 8.90	20.17 ± 13.86	0.257	22.64 ± 10.25	23.35 ± 9.96	24.25 ± 9.20	24.34 ± 10.61	0.632
Fruits (g/day)	222.36 ± 155.12	285.01 ± 209.66	378.33 ± 309.14	589 ± 425.33	< 0.001	363.3 ± 27.4	423.75 ± 22.12	436.43 ± 12.27	517.2**3** ± 14.58	< 0.001
Vegetables (g/day)	331.35 ± 181.87	276.24 ± 179.77	432.39 ± 283.76	648.36 ± 402.08	< 0.001	345.23 ± 7.5	355.45 ± 10.42	362.35 ± 8.61	379.46 ± 9.86	< 0.001
Red meat (g/day)	41.83 ± 16.14	35.8 ± 26.22	52.51 ± 58.34	61.81 ± 89.84	< 0.001	35.84 ± 18.19	33.83 ± 16.21	49.56 ± 17.36	58.84 ± 18.84	< 0.001
White meat (g/day)	53.33 ± 22.58	58.13 ± 44.36	75.87 ± 58.52	93.35 ± 109.54	< 0.001	34.21 ± 21.53	52.35 ± 21.29	74.37 ± 21.61	82.11 ± 21.36	< 0.001
Dairy (g/day)	363.87 ± 195.34	345.56 ± 159.33	534.22 ± 366.63	756.38 ± 463.89	< 0.001	311.12 ± 117.54	327.8 ± 119.63	329.19 ± 118.41	394.68 ± 121.69	< 0.001
Legumes (g/day)	28.82 ± 3.12	22.35 ± 2.37	35.6 ± 4.27	54.6 ± 5.83	< 0.001	48.21 ± 4.15	55.61 ± 5.34	56.95 ± 5.17	60.74 ± 6.29	< 0.001
Nuts (g/day)	10.47 ± 2.58	13.72 ± 2.82	20.1 ± 5.13	26.8 ± 4.28	< 0.001	13.39 ± 1.35	12.92 ± 1.65	14.19 ± 1.45	22.21 ± 1.74	< 0.001
Grains (g/day)	230 ± 90.6	352.23 ± 121.37	440.28 ± 152.42	668.19 ± 159.34	< 0.001	248.59 ± 85.83	255.8 ± 89.26	256.63 ± 92.12	344.91 ± 120.47	< 0.001
Salt (g/day)	6.26 ± 2.47	7.34 ± 2.62	8.76 ± 2.37	9.81 ± 2.25	< 0.001	5.62 ± 2.14	6.14 ± 2.38	7.27 ± 2.31	9.71 ± 3.17	< 0.001
Calcium (mg/d)	1428.33 ± 1374.32	1322.98 ± 1035.47	1517.33 ± 1421.52	1675.14 ± 1452.27	0.673	1366.18 ± 1171.28	1453.37 ± 1151.16	1474.2 ± 1153.56	1597.5 ± 1185.33	0.547
Magnesium (mg/d)	262.19 ± 128.38	282.31 ± 112.37	287.22 ± 125.37	396.11 ± 123.47	0.254	210.5 ± 124.61	274.5 ± 123.35	269.6 ± 119.42	353.10 ± 134.83	0.324
SFA (g/d)	27.11 ± 14.18	22.82 ± 11.34	29.63 ± 11.74	32.87 ± 12.78	< 0.001	30.21 ± 12.43	29.93 ± 11.24	29.51 ± 11.32	30.98 ± 12.39	0.217
PUFA (g/d)	18.2 ± 8.33	15.62 ± 8.12	19.21 ± 6.34	21.59 ± 7.29	< 0.001	23.10 ± 6.92	23.16 ± 7.29	25.19 ± 8.10	26.53 ± 8.91	0.741

Abbreviations: MUFA, monounsaturated fatty acid; SFA, saturated fatty acid.

^a^ *p* values resulted from ANOVA test. Data are mean ± SD obtained from ANOVA. *p* value < 0.05 indicates significant level.

**TABLE 5:** Energy, nutrient, and food group intakes of study participants across quartiles of the dietary glycemic index (DGI).

Variable	Case (120)					Control (250)				
	Q1	Q2	Q3	Q4	*p* ^a^	Q1	Q2	Q3	Q4	*p* ^a^
Energy (Kcal/day)	2180.27 ± 425.32	2457.45 ± 682.37	2628.69 ± 787.26	2713.31 ± 793.12	< 0.001	2324.12 ± 507.31	2361.34 ± 509.65	2449.37 ± 695.24	2943.12 ± 684.17	< 0.001
Carbohydrate (g/day)	293.21 ± 75.65	351.35 ± 110.33	350.64 ± 121.34	397.22 ± 131.82	< 0.001	354.32 ± 114.72	388.15 ± 125.31	398.21 ± 110.77	440.64 ± 114.34	< 0.001
Fat (g/day)	75.72 ± 18.12	78.5 ± 29.7	84.34 ± 25.57	91.87 ± 35.74	< 0.001	86.22 ± 30.61	89.59 ± 34.37	92.32 ± 35.95	97.34 ± 34.42	< 0.001
Protein (g/day)	68.21 ± 23.1	76.3 ± 29.7	79.8 ± 36.4	88.5 ± 24.54	< 0.001	82.18 ± 22.34	86.51 ± 25.31	92.31 ± 26.41	98.23 ± 28.75	< 0.001
Total fiber (g/day)	18.2 ± 10.21	19.1 ± 9.34	20.12 ± 8.20	29.28 ± 13.34	< 0.001	25.34 ± 9.14	26.67 ± 10.23	26.34 ± 11.29	28.36 ± 12.31	0.632
Fruits (g/day)	236.31 ± 160.22	264.11 ± 172.14	395.18 ± 221.37	629.79 ± 240.18	< 0.001	386.22 ± 29.34	395.36 ± 32.34	400.37 ± 33.75	450.36 ± 35.47	< 0.001
Vegetables (g/day)	297.36 ± 143.76	328.15 ± 227.52	391.78 ± 285.32	538 ± 291.43	< 0.001	388.36 ± 17.68	394.62 ± 18.70	402.64 ± 18.61	498.22 ± 19.62	< 0.001
Red meat (g/day)	35.42 ± 19.35	36.14 ± 20.13	42.34 ± 25.34	64.27 ± 29.31	< 0.001	38.92 ± 21.36	39.12 ± 22.34	45.29 ± 25.52	57.21 ± 25.31	< 0.001
White meat (g/day)	45.38 ± 29.62	55.35 ± 43.28	73.79 ± 61.62	90.25 ± 68.37	< 0.001	40.25 ± 21.63	53.75 ± 22.63	76.39 ± 23.74	82.35 ± 23.37	< 0.001
Dairy (g/day)	262.75 ± 129.34	395.75 ± 221.43	492.15 ± 322.31	873 ± 395.12	< 0.001	332.62 ± 115.37	342.34 ± 120.67	375.63 ± 119.63	398.62 ± 125.70	< 0.001
Legumes (g/day)	26.13 ± 2.45	29.64 ± 3.38	38.82 ± 4.32	48.1 ± 5.24	< 0.001	50.31 ± 3.96	56.45 ± 5.42	58.67 ± 5.28	65.17 ± 6.22	< 0.001
Nuts (g/day)	9.25 ± 1.04	10.24 ± 1.21	18.48 ± 3.35	25.25 ± 5.18	< 0.001	14.42 ± 1.96	15.35 ± 1.21	16.37 ± 1.67	21.34 ± 1.96	< 0.001
Grains (g/day)	219.24 ± 85.13	361.54 ± 75.32	442.37 ± 78.34	576 ± 75.17	< 0.001	250.14 ± 78.36	257.37 ± 80.38	289.55 ± 95.19	356.41 ± 119.10	< 0.001
Salt (g/day)	6.19 ± 2.64	8.23 ± 3.80	9.02 ± 2.25	9.67 ± 2.21	< 0.001	5.37 ± 2.85	5.28 ± 2.10	7.15 ± 2.68	8.96 ± 2.72	< 0.001
Calcium (mg/d)	1173.21 ± 1157.39	1431.28 ± 1156.31	1721.38 ± 1552.39	1521.32 ± 1320.38	< 0.001	1398.27 ± 112.35	1465.28 ± 1129.63	1495.27 ± 1141.20	1516.45 ± 1109.33	0.863
Magnesium (mg/d)	234.16 ± 110.31	254.29 ± 112.35	265.31 ± 121.18	295.21 ± 119.65	< 0.001	208.87 ± 111.141	274.5 ± 123.35	269.6 ± 119.42	353.10 ± 134.83	0.563
SFA (g/d)	24.22 ± 5.22	28.1 ± 6.54	29.82 ± 6.65	32.52 ± 6.32	< 0.001	32.68 ± 11.37	30.52 ± 12.10	28.63 ± 13.39	33.67 ± 14.42	0.137
PUFA (g/d)	16.25 ± 3.25	18.15 ± 4.68	18.72 ± 4.29	20.25 ± 4.63	< 0.001	24.12 ± 6.85	25.31 ± 7.12	27.20 ± 8.31	27.21 ± 8.51	0.320

Abbreviations: MUFA, monounsaturated fatty acid; SFA, saturated fatty acid.

^a^ *p* values resulted from ANOVA test. Data are mean ± SD obtained from ANOVA. *p* value < 0.05 indicates significant level.

The association between the higher dietary GI and GL and the odds of DOR is reported in Table 6. In the crude model and the model adjusted for physical activity and energy intake, there was no significant relationship between the studied indices and the odds of DOR. However, in Model II, after controlling for physical activity, energy intake, FM, and BMI, women in the highest quartile of GL and GI score had a 13% (OR 1.13; 95% CI 1.07–2.68) and 19% (OR 1.19; 95% CI 0.59–1.87, *p* = 0.038) increased odds of DOR, respectively, compared to those in the first quartile.

**TABLE 6:** Odds ratio (95% CI) of DOR according to quartiles of GL and GI.

	GL					GI				
	Q1	Q2	Q3	Q4	*p*‐trend	Q1	Q2	Q3	Q4	*p*‐trend
DOR/Control	31/61	27/60	30/65	32/64		31/64	30/63	29/60	30/63	
Crude	Ref (1.00)	0.88 (0.47–1.65)	0.90 (0.49–1.67)	0.98 (0.82–1.80)	0.981	Ref (1.00)	0.96 (0.53–1.81)	0.99 (0.53–1.84)	1.02 (0.53–1.83)	0.970
Model 1	Ref (1.00)	0.89 (0.38–1.52)	0.88 (0.45–1.71)	1.09 (0.88–2.15)	0.622	Ref (1.00)	0.98 (0.55–1.59)	0.97 (0.59–1.82)	1.07 (0.55–1.85)	0.504
Model 2	Ref (1.00)	0.91 (0.35–1.50)	1.00 (0.49–2.04)	1.13 (1.07–2.68)	0.042	Ref (1.00)	0.99 (0.57–1.63)	1.03 (0.61–1.85)	1.19 (0.59–1.87)	0.038

*Note:* Multivariable logistic regression models were used with adjustment of potential confounders. Model 1: Physical Activity + Energy intake (kcal/day). Model 2: Model 1 + Fat Mass (kg) and Body mass index.

Abbreviation: DOR, diminished or decreased ovarian reserve.

## 4 Discussion

To our knowledge, this is the first to explore the link of carbohydrate composition with the odds of DOR. In this case–control study, significant associations were found between the highest quartile of dietary glycemic indices and the odds of DOR, independent of the effect of confounding variables. Besides, both GI and GL were directly associated with AFC concentration. However, the link of carbohydrate quantity and quality with serum AMH levels was evident only in the control group.

Generally, DOR, which is characterized by a reduction in both the quantity and quality of oocytes, has the potential to lead to early menopause and poor fertility outcomes (Nikolaou and Templeton 2003). In spite of the fact that the etiology of DOR is still vague, a growing body of evidence has shown that, in addition to age and genetics, psychological stress, smoking, and environmental factors, particularly diet and exercise patterns, contributed to its development (Zhu et al. 2023). Interestingly, diet, and carbohydrates in particular, have been hypothesized to affect fertility (Chavarro et al. 2009), while the precise mechanisms behind this association are not fully elucidated.

Regardless of the lack of data on the link of dietary glycemic indices with ovarian reserve, several previous investigations have addressed the influences of dietary factors on fecundability and ovulation disorders (Maldonado‐Cárceles et al. 2020). In this regard, special attention has been paid to vitamin D and soy products in the context of fertility (Moridi et al. 2020; Mitsunami et al. 2023). Besides, adherence to certain dietary patterns has been revealed to be associated with improvements in measures of ovarian reserve (Eskew et al. 2022). For instance, a cross‐sectional analysis of cohort data revealed a positive relation between improved markers of ovarian reserve and a higher compliance with profertility diet (PFD) in overweight and obese women (Eskew et al. 2022). It is worth noting that this dietary pattern (PFD) was defined by higher consumption of whole grains, soy and seafood, dairy, supplemental folic acid, vitamin B12, vitamin D, and low pesticide fruits and vegetables (Gaskins et al. 2019). Moreover, particular benefits of the Mediterranean diet (MD) on female reproductive functions have been documented previously, whereas its direct relations with ovarian reserve have not been definitively established (Toledo et al. 2011).

The current analysis indicated that dietary glycemic indices were positively related to AFC concentration, as a reliable predictor of ovarian reserve. However, the AMH‐glycemic indicators relations were observed only in the control group. Consistent with our findings, a higher intake of whole grains, which tend to have lower GI values (Qi and Hu 2007), as one of the components of PFD, increased AFC and AMH levels in obese women, as reported in the study by Eskew et al. (2022). Likewise, data from a cohort NHS II study showed positive relations of total carbohydrate intake and dietary GL with anovulatory infertility among premenopausal women without a history of infertility (Chavarro et al. 2009). Besides, sweetened, carbonated beverages have been speculated to be a stronger reproductive toxicant than caffeine intake (Machtinger et al. 2017). Conversely, the results of the Sister Study cohort revealed strong positive links between the serum AMH concentration and dietary GL and total carbohydrate intake (Anderson, Mark Park, et al. 2018) whereas they argued that these observed associations may be due to an AMH‐lowering influence of dietary fat. In other words, in their study, the proportion of energy from carbohydrate and fat intake was inversely correlated. Accordingly, some human and experimental evidence has shown that exposure to high‐fat diets may compromise fertility (Hohos and Skaznik‐Wikiel 2017; Skaznik‐Wikiel et al. 2016).

An increasing body of evidence has shown an association of a lower ovarian reserve with a greater vulnerability to CVD (de Kat et al. 2017). Accordingly, AMH, as the most promising determinant of ovarian reserve, has a physiological role beyond the scope of the female reproductive system (de Kat et al. 2015). Currently, it has been suggested that AMH levels and its decline rate can be a probable contributor to CVDs and coronary heart disease among women independent of menopausal condition and metabolic risk factors (de Kat et al. 2017). However, the results of studies seeking the linkage between this marker and metabolic syndrome and cardiometabolic risk factors are heterogeneous (de Kat et al. 2015; Bleil et al. 2013). These inconclusive results may be partially explained by the varied nature of the study designs, sample size, and population setting, which limit an overall interpretation.

Taken together, considering the possible role of AMH levels in the pathophysiology of chronic conditions, finding nutritional determinants of ovarian reserve decline for primary and secondary prevention of these health consequences is a public health priority. Despite the scarcity of the literature regarding the probable influences of diet on ovarian reserve (Moslehi et al. 2019; Chavarro et al. 2009) and the lack of research exploring the association of dietary glycemic measures with DOR and its biomarkers, a number of studies have documented the metabolic relevance of dietary glycemic indices (Eslamian et al. 2017). In this regard, the beneficial effects of low glycemic diets on cardiometabolic and reproductive outcomes in women with polycystic ovary syndrome (PCOS) have been evidenced in some recent systematic reviews and meta‐analyses (Kazemi et al. 2021; Saadati et al. 2021). Similarly, some reports have confirmed the robust associations of GI and GL with the odds of diabetes development and incidence (Teymoori et al. 2021). Nevertheless, there are some epidemiological studies that have yielded opposite findings (Vega‐López et al. 2018). These discrepancies may be due, in part, to wide variations in population characteristics (ethnicity, BMI, etc.), dietary assessment techniques, and varieties in reported GI values for some comparable food items along with intra‐ and interindividual variation in glucose responses to specific foods.

The potential biological mechanisms and pathways by which high dietary glycemic indicators negatively influence fertility and ovarian function may result from their impacts on insulin resistance and inflammation or oxidative stress (Silvestris et al. 2019). Some reports have proposed that hyperinsulinemia following high glycemic diets may contribute to hyperandrogenism and higher levels of insulin‐like growth factor I (IGF‐I) which can eventually lead to ovulation abnormalities and endocrine disturbances (Silvestris et al. 2019). Importantly, inflammation and oxidative damage have been shown to be adversely related to a higher speed of ovarian reserve decline (Barrea et al. 2018). Notably, earlier studies have supported the possibility that diets with high glycemic measures and low fiber content may exert proinflammatory effects (Levitan et al. 2008) although the precise relationship remains debatable (Milajerdi et al. 2018). On the other hand, conclusive evidence has been found regarding favorable effects of dietary antioxidant compounds against DOR mainly through lowering the production of reactive oxygen species in the ovaries (Xu et al. 2018). Moreover, excess adiposity induced by high GI/GL diets has a negative influence on ovarian function and fertility since lower AMH and AFC levels have been reported in obese women compared to those with normal weight (Prieto‐Huecas et al. 2023).

The major strength of the current study is that it is the first attempt to relate dietary carbohydrate quantity and quality to DOR odds in women. Matching by age and BMI was conducted in this case–control study to eliminate confounding. A large sample size, using valid and reliable tools for gathering data and controlling for several covariates in analyses, were other unique features of our study. Nonetheless, there are several limitations that influence the interpretation of our findings and require consideration. First, owing to the retrospective nature of this study, causality cannot be inferred. Second, selection bias and recall bias in this type of study may lead to an inaccurate estimate of association. Third, GI/GL from FFQ may not properly predict the glycemic responses of the single foods or mixed meals. Fourth, despite several potential confounders being adjusted, the effects of residual confounding variables, including psychological and genetic factors, age at first pregnancy, number of births, and other unknown confounders, could not be completely excluded. Fifth, the generalizability of our findings to women in the general population is a concern, as this study was performed among women attending an infertility clinic. Considering the aforementioned drawbacks, further studies with a prospective cohort design involving a more diverse sample of reproductive‐aged women from the general population are warranted to better establish the relationship of interest. Besides, using more precise dietary assessment tools such as weighed food records and multiday 24‐h dietary recalls may provide the detailed information necessary for accurately computing GI and GL. Moreover, the biological plausibility of the observed associations should be explored through mechanistic studies demonstrating how high‐glycemic diets influence follicular development, oxidative stress, and ovarian inflammation. Additionally, using consistent and standardized diagnostic criteria for DOR can improve comparability across studies.

In summary, our results support the hypothesis that greater intakes of high‐GI/GL foods may be associated with increased odds of DOR and its related biomarkers in women. Hence, since DOR is a recognized contributor to infertility, our findings suggest that the modulation of dietary carbohydrate intake (e.g., adopting lower‐GI foods) can be regarded as a noninvasive, lifestyle‐based approach to help preserve ovarian reserve and potentially postpone fertility decline. Accordingly, personalized dietary recommendations according to glycemic response could be integrated into comprehensive strategies for managing fertility. However, due to diet contribution to ovarian reserve being still in its infancy, additional studies, especially of prospective and clinical trial design, are required to confirm our results.

## Author Contributions

**Abed Ghavami:** data curation (equal), formal analysis (equal), software (equal). **Mahdieh Khodarahmi:** writing – review and editing (equal). **Sanaz Mehrabani:** writing – original draft (equal). **Amin Mokari‐Yamchi:** writing – original draft (equal). **Hatav Ghasemi‐Tehrani:** investigation (equal), methodology (equal). **Mahdi Vajdi:** conceptualization (equal), data curation (equal), formal analysis (equal), methodology (lead), resources (equal), supervision (equal), writing – original draft (lead), writing – review and editing (equal). **Gholamreza Askari:** conceptualization (equal), data curation (equal), investigation (equal).

## Ethics Statement

The studies involving humans were approved by the Local Ethics Committee of Isfahan University of Medical Sciences (IR.ARI.MUI.REC.1401.297). The studies were conducted in accordance with local legislation and institutional requirements. The participants provided their written informed consent to participate in this study.

## Consent

All patients agreed to participate in the study and signed the informed consent form.

## Conflicts of Interest

The authors declare no conflicts of interest.

## Data Availability

The original contributions presented in the study are included in the article/Supporting Information; further inquiries can be directed to the corresponding author.
